# New real-time patient radiation dosimeter for use in radiofrequency catheter ablation

**DOI:** 10.1093/jrr/rry110

**Published:** 2019-01-08

**Authors:** Mamoru Kato, Koichi Chida, Masaaki Nakamura, Hideto Toyoshima, Ken Terata, Yoshihisa Abe

**Affiliations:** 1 Department of Radiology and Nuclear Medicine, Research Institute for Brain and Blood Vessels – Akita, 6–10 Senshu-Kubota Machi, Akita, Akita, Japan; 2 Course of Radiological Technology, Health Sciences, Tohoku University Graduate School of Medicine, 2-1 Seiryo-cho, Sendai, Miyagi, Japan; 3 Department of Cardiology, Division of Internal Medicine, Research Institute for Brain and Blood Vessels – Akita, 6–10 Senshu-Kubota Machi, Akita, Akita, Japan

**Keywords:** real-time dosimeter, patient dose, radiophotoluminescence glass reference dosimeter (RPLD), radiation skin dose, peak skin dose, photoluminescence sensor

## Abstract

In a previous study, we reported on a novel (prototype) real-time patient dosimeter with non-toxic phosphor sensors. In this study, we developed new types of sensors that were smaller than in the previous prototype, and clarified the clinical feasibility of our newly proposed dosimeter. Patient dose measurements obtained with the newly proposed real-time dosimeter were compared with measurements obtained using a calibrated radiophotoluminescence glass reference dosimeter (RPLD). The reference dosimeters were set at almost the same positions as the new real-time dosimeter sensors. We found excellent correlations between the reference RPLD measurements and those obtained using our new real-time dosimeter (*r*^2^ = 0.967). However, the new type of dosimeter was found to underestimate radiation skin dose measurements when compared with an RPLD. The most probable reason for this was the size reduction in the phosphor sensor of the new type of dosimeter. We believe that, as a result of reducing the phosphor sensor size, the backscattered X-ray irradiation was underestimated. However, the new dosimeter can accurately determine the absorbed dose by correcting the measured value with calibration factors. The calibration factor for the new type dosimeter was determined (by linear regression) to be ~1.15. New real-time patient dosimeter design would be an effective tool for the real-time measurement of patient skin doses during interventional radiology treatments.

## INTRODUCTION

Although numerous patients have greatly benefited from cardiac interventional radiology (IR), radiation-induced skin injuries have been reported in several cardiac IR procedures [1–12]. These deterministic effects arise because these complicated procedures tend to require an extended fluoroscopic exposure time and repeated digital angiography (DA) with various angles of X-ray projection. In addition, these procedures often need to be performed repeatedly. Thus, in order to increase the efficacy and safety of cardiac IR procedures, one must therefore reduce the patient’s exposure to excessive X-ray treatments. In addition, the real-time monitoring of patient radiation doses is essential, and in particular, the peak radiation skin dose (PSD) should be monitored in real-time to avoid radiation-induced skin injury [1, 13–25].

Recently, the International Commission on Radiological Protection (ICRP) emphasized the necessity of using diagnostic reference levels (DRLs) to optimize patient doses and recommended that, during actual IR procedures, the maximum cumulative absorbed dose in the skin should be monitored in real-time [25]. However, although the real-time monitoring of patient doses is important, there are few feasible real-time dosimeters currently available for IR patients [26].

**Fig. 1.**
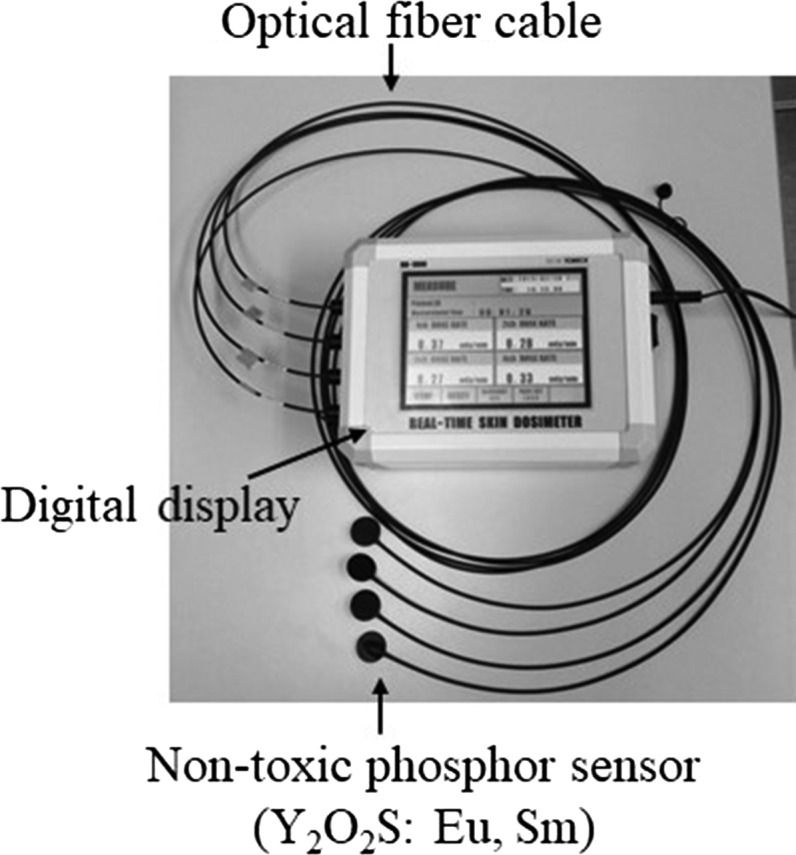
New type of real-time patient dosimeter.

In a previous study, we reported on a novel (prototype) real-time patient dosimeter with four non-toxic phosphor sensors suitable for red-emission phosphor in the medical X-ray range [27–29] (Fig. 1). We conducted a feasibility study in a clinical setting to investigate the use of the prototype dosimeter during a number of different procedures [coronary angiography (CAG), radiofrequency catheter ablation (RFCA), and percutaneous coronary intervention (PCI)].

**Fig. 2.**
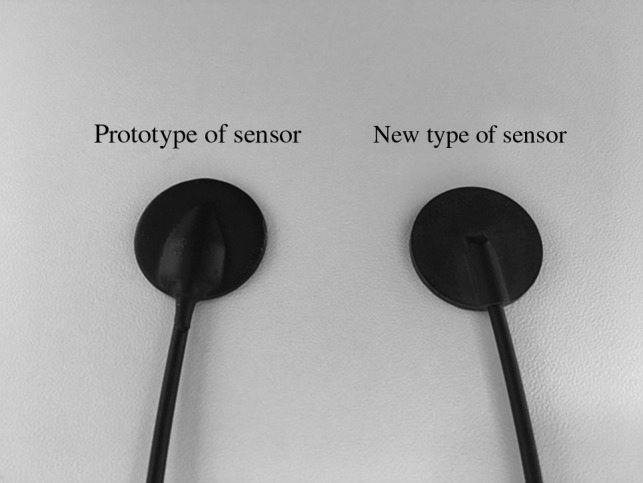
Non-toxic phosphor sensor (Y_2_O_2_S: Eu, Sm).

In this study, we developed new types of sensors that were smaller than in the previous prototype (Fig. 2) and measured the patient skin absorbed dose in RFCA treatments. RFCA is a standard treatment modality for tachyarrhythmia and is performed to disconnect the pathway involved in the abnormal rhythm. Significantly, RFCA is an effective procedure for adults as well as children; however, RFCA tends to be a complex procedure, which increases the fluoroscopic time and can deliver high radiation doses to the patient. Moreover, the patient’s applied radiation dose depends upon the type of arrhythmia they exhibit.

## MATERIALS AND METHODS

### Patients

This study included 40 RFCA procedures and was conducted at the Research Institute for Brain and Blood Vessels – Akita (Akita, Japan). The patients were selected at random and included 26 males and 14 females (Table 1). This was a single-institution study and was approved by the local Committee on Human Research.

**Table 1.** Summary of the patient characteristics (mean ± standard deviations where applicable) for radiofrequency catheter ablation (*n* = 40)

Age (years)	Height (cm)	Weight (kg)	Male/Female	Fluoroscopy time (min)	Cumulative air kerma (mGy)	Kerma area product (Gy · cm^2^)
59.5 ± 14.4	164.7 ± 11.4	61 ± 12.9	26/14	25.3 ± 15.5	421.6 ± 428.8	59.9 ± 50.3

This study was conducted in accordance with the Declaration of Helsinki. All protocols were approved by Research Institute for Brain and Blood Vessels – Akita (Akita, Japan). Informed consent was obtained from patients before commencing procedures.

### X-ray equipment

The main angiography X-ray unit used in this study was a digital cine single-plane system (Artis Zee FA; Siemens Healthcare, Germany) with a 32-cm mode flat-panel detector. Digital cine acquisition was performed at 7.5 frames/s, and pulsed fluoroscopy was performed at 7.5 pulses/s. Four standard tube angulations were used in our clinical RFCA setting: right anterior oblique (RAO) 60° view, RAO 30° view, antero–posterior (AP) view, and left anterior oblique (LAO) 50° view.

### Dosimetry

Our new smaller real-time dosimeter comprised photoluminescence sensors (non-toxic phosphor, maximum of four sensors), an optical fiber cable, a photodiode, and a digital display that included the power supply [28]. The new sensor used the same Y_2_O_2_S: Eu, Sm phosphor material as was used for the prototype, since we note that Y_2_O_2_S: Eu, Sm is non-toxic and exhibits relatively high sensitivity. The photoluminescence sensor size of prototype was a square of 2.5 × 2.5 mm and the photoluminescence sensor size of new type was a circle of radius 0.7 mm. The sensor area of new type was reduced to 1/4.

Patient dose measurements obtained with the newly proposed real-time dosimeter were compared with measurements obtained using a calibrated radiophotoluminescence glass reference dosimeter (RPLD) [30–32]. The measurement/readout system used for the RPLD (GD-302 M sensor; Asahi Techno Glass, Tokyo, Japan) was the DoseAce FGD-1000 unit (Chiyoda Technol Corp., Tokyo, Japan). The calibration factor for the RPLD used in this study was determined to be 0.348, based on a calibration experiment conducted in air using a thimble-type 6-ml ion-chamber (model-9015; Radcal Corp., Monrovia, CA, USA; traceable from the national standard exposure dose).

We previously reported on a modified dosimetry gown that can measure the entrance skin dose during PCI using multiple RPLDs [31]. From the study, we created the entrance skin dose distribution map of patients, and we could comprehend the irradiation field of each angle. The measured radiation skin dose was examined using the four sensors of the new real-time dosimeter, arbitrarily positioned at the fields of the RFCA working angle. The reference dosimeters were set at almost the same positions (within 1 cm) of the new real-time dosimeter sensors. The RPLD and the new real time dosimeter were responsive to backscattered radiation, and the measurement doses were entrance surface dose (air kerma). In our clinic, the entrance surface dose was transformed to skin absorbed dose based on the ‘Tables of X-Ray Mass Attenuation Coefficients’ by S. M. Seltzer and J. H. Hubbell [33]. We also interviewed physicians regarding the visibility (i.e. whether the view was impeded) of the radiographic images in which the new dosimeter sensors were visible. Finally, the relationship between the reference and the new type of dosimeter measurements was analyzed using linear regression.

## RESULTS

**Fig. 3.**
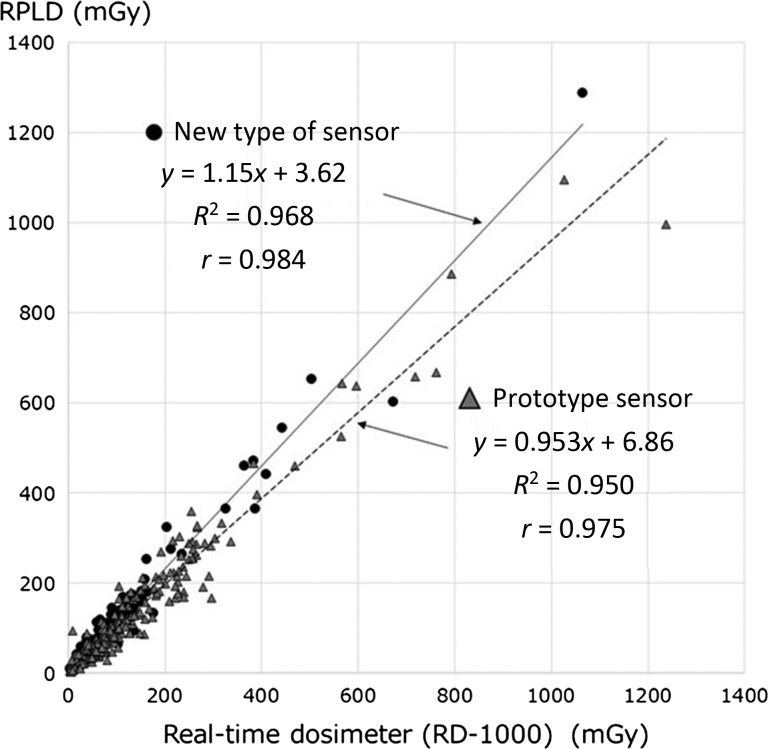
Relationship between the reference dosimeter (radiophotoluminescence glass dosimeter; RPLD) measurements of the patient radiation dose and those of the new type of dosimeter in a clinical setting.

Figure 3 shows the correlations between the RPLD measurements and the real-time dosimeter measurements. The prototype sensor and the new type of sensor have excellent correlation coefficients between the RPLD measurements. From the linear regression fit, *y* = 1.15*x* + 3.62 (*r*^2^ = 0.968), the new type of dosimeter is seen to underestimate the dose by 15% with respected to the calibrated reference RPLD dosimeter.

**Fig. 4.**
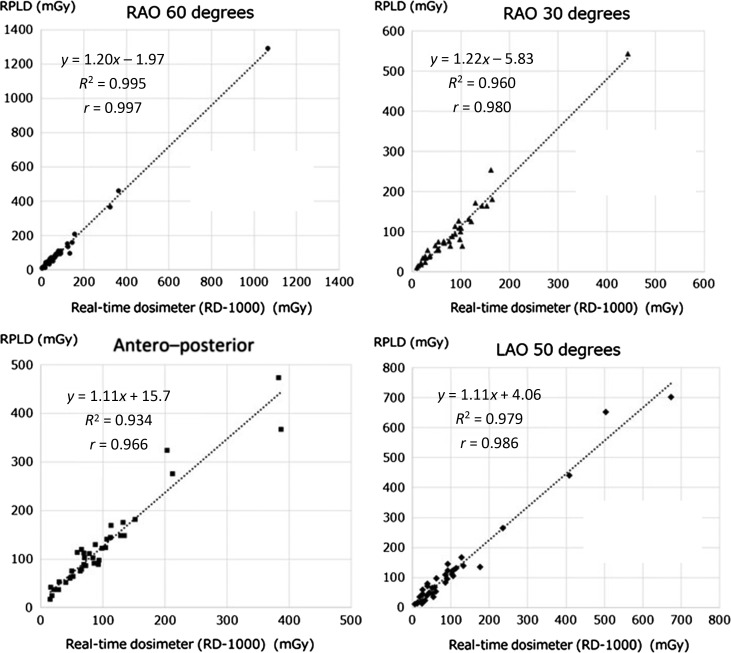
Angular dependence of new real-time dosimeter. RAO = right anterior oblique, LAO = left anterior oblique.

Figure 4 shows the Pearson correlation test revealed statistically positive correlations between the measurements of each irradiation field obtained using the RPLDs and those measurements obtained using the new type of sensor, RAO60 (*r* = 0.997, *P* < .01), RAO30 (*r* = 0.980, *P* < .01), P-A (*r* = 0.966, *P* < .01) and LAO50 (*r* = 0.986, *P* < .01), respectively. Thus, we concluded that new real-time dosimeter had no angular dependence.

**Fig. 5.**
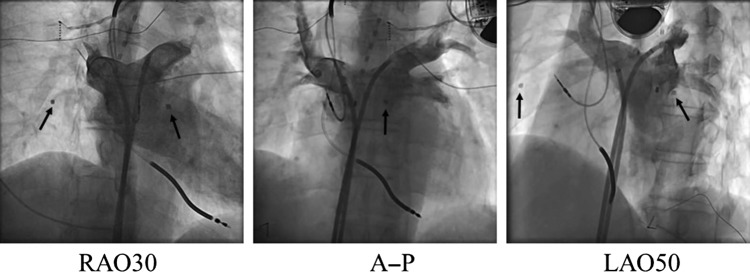
Angiogram taken during a patient’s dose measurement in an RFCA procedure. Arrows indicate the locations of the prototype dosimeter sensors. The size of the photoluminescence sensor was a square of 2.5 × 2.5 mm. RFCA = radiofrequency catheter ablation, RAO = right anterior oblique, LAO = left anterior oblique.

**Fig. 6.**
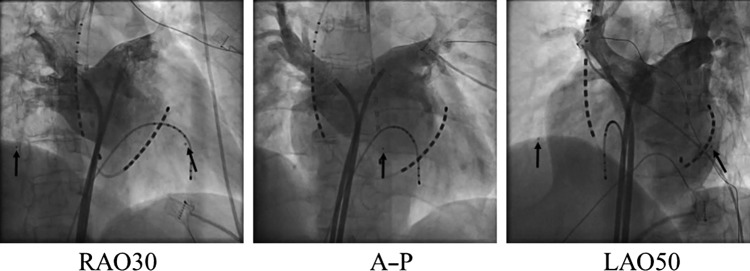
Angiogram taken during a patient’s dose measurement in an RFCA procedure. Arrows indicate the locations of the new type of dosimeter sensors. The size of the photoluminescence sensor was a circle of radius 0.7 mm. RFCA = radiofrequency catheter ablation, RAO = right anterior oblique, LAO = left anterior oblique.

Figures 5 and 6 show an angiogram taken during a patient’s dose measurement in an RFCA procedure. Although the prototype dosimeter sensor (Fig. 5) is quite noticeable, our new smaller dosimeter sensor (Fig. 6) is only slightly noticeable. In both cases, the dosimeter cable is invisible in the angiographic image, in addition to the reference RPLDs. We note that the number of complaints received from physicians and other medical staff regarding obstruction caused by the new type of sensors during the procedure was less than the number received for the original prototype dosimeter sensor.

## DISCUSSION

According to ICRP Publication 105, DRLs for IR are used to promote the management of patient doses with a view to avoiding stochastic effects. In addition, the document recommends that the PSD be measured during actual IR procedures to avoid deterministic effects.

This study was performed to clarify the clinical feasibility of our new type of patient dosimeter. We found an excellent correlation between the patient skin dose as measured by a reference RPLD and as measured by our new type of dosimeter in a clinical setting (*r^2^* = 0.968). Although both our original prototype sensor and the new sensor are slightly visible on clinical images, the new sensor is significantly smaller than the prototype sensor. Therefore, the new sensor dose not impact the coronary artery images significantly. We conclude that the newly proposed dosimeter is also feasible for clinical use in CAG and PCI procedures.

However, the new type of dosimeter was found to underestimate radiation skin dose measurements when compared with a calibrated reference RPLD dosimeter. The most probable reason for this was a reduction in the phosphor sensor size of the new type of dosimeter. As a result of reducing the phosphor sensor size, we expect that the backscatter X-ray was underestimated, and that the collection efficiency between the phosphor sensor and the optical fiber cable decreased. However, the new dosimeter can measure an accurate (i.e. in accordance with the reference RPLD dosimeter) absorbed dose by correcting the value with calibration factors. The calibration factor of the new type dosimeter was determined by linear regression to be ~1.15.

Some differences were also found between the reference RPLD measurements and the new type of dosimeter measurements. One probable reason for these differences is that the new sensor type is angle-dependent. Although both sets of sensors were located at approximately the same positions, there was a small distance (~1 cm) between the sensor positions. This means that, in some cases, one of the sensors (i.e. the RPLD or our new dosimeter) may not have been located within the primary X-ray beam for some of the projections used during each procedure.

In conclusion, we found excellent correlations between the reference radiation skin dose measurements and those obtained using our newly proposed real-time dosimeter (*r*^*2*^ = 0.968). Therefore, our new device can be used to accurately measure radiation skin doses at the specified measuring points in a clinical setting using a calibration factor of 1.15.

Moreover, the sensor and cable of the proposed dosimeter do not interfere with the IR procedure. As a result, we believe that our new photoluminescence sensor dosimeter design would be an effective tool for the real-time measurement of patient skin doses during IR treatments.
